# Anaplastic lymphoma kinase-positive pulmonary inflammatory myofibroblastic tumour: a case report

**DOI:** 10.1186/s13256-024-04472-9

**Published:** 2024-04-10

**Authors:** Daniel Tong, Julia Chisholm, Brendan Madden, Merina Ahmed

**Affiliations:** 1 Lung Unit, The Royal Marsden NHS Foundation Trust, Sutton, UK; 2 Children and Young People’s Unit, Institute of Cancer Research, Royal Marsden Hospital, Sutton, SM2 5NG UK; 3 Department of Cardiothoracic Medicine, St Georges Hospital, Blackshaw Road, London, SW17 0QT UK

**Keywords:** Pulmonary inflammatory myofibroblastic tumour, ALK, Multimodality, Radiotherapy and pulse steroids, Long-term remission

## Abstract

**Background:**

Pulmonary inflammatory myofibroblastic tumour (IMT) is a rare condition that usually presents in young individuals and is associated with anaplastic lymphoma kinase (ALK)-translocation.

**Case presentation:**

We report a case of an 18-year-old Caucasian man with ALK-translocated pulmonary IMT treated with multimodality therapy. The patient presented with breathlessness and was found to have a collapsed left lung. Further investigations revealed an ALK-translocated pulmonary IMT. This is usually treated with an ALK-inhibitor but patient declined after discussing potential side-effects and had repeated rigid bronchoscopic interventions for local disease control. Due to persistent local recurrence, patient received radical external beam radiotherapy (EBRT) with pulse steroids, and one year later started on Ibuprofen, a non-steroidal anti-inflammatory agent (NSAID). Following multimodality treatment, he developed a complete response. He remains treatment-free for the past seven years. Eleven years on from his diagnosis, he remains in remission with a ECOG performance status of zero.

**Conclusions:**

Achieving long-term local control in pulmonary IMT can be challenging. Multimodality treatment is sometimes needed but the overall outlook remains good.

## Background

Inflammatory myofibroblastic tumour (IMT) is a rare tumour of myofibroblastic spindle cells with inflammatory infiltrates [1]. There is no standardised staging specific to IMT. It is classified as having an intermediate malignant potential, with a propensity for local recurrence but metastasis is rare [2]. It is also referred to as plasma cell granuloma, and formerly fell under the category of inflammatory pseudotumour, which included neoplastic and non-neoplastic processes [2]. It often presents in young individuals in both pulmonary and extrapulmonary sites, and anaplastic lymphoma kinase (ALK) translocation is found in 47% of individuals [1–3]. Standard treatment option is surgical resection, and re-excision on recurrence [4, 5]. Inoperable tumours or cases with incomplete resection can be treated with a variety of treatment options, including corticosteroids, non-steroidal anti-inflammatory agents (NSAIDs), radiotherapy, adjuvant chemotherapy or ALK-inhibitors when ALK translocation is present [6–11].

We describe a case of an 18-year-old with ALK-positive pulmonary IMT treated with multimodal local therapy and achieving long-term remission (11 years since his diagnosis). He had disease resistant to repeated rigid bronchoscopic dilatation and laser therapy and did not wish to consider systemic treatments due to their potential side-effects. He was trailed on corticosteroids but did not tolerate it. He later received radical radiotherapy with pulse steroids, and was started on NSAIDs one year later due to clinical deterioration. He had complete response and has remained off-treatment for the last seven years. He continues to have an ECOG performance status of zero. This is the first case report of pulmonary IMT treated with radical radiotherapy and pulse steroids.

## Case presentation

### Clinical presentation and diagnosis

An 18-year-old Caucasian man presented to his local hospital with acute shortness of breath (*T* = 0). Physical examination showed marked reduction in air entry to the left lung. Chest X-ray showed left lung collapse.

Five years prior to this, he started experiencing episodic breathlessness and was eventually diagnosed with asthma. He was initiated on inhalers which did not provide significant symptomatic relief. During this period, he had episodes of bronchitis, about twice a year, which responded to antibiotics. Up until his acute presentation to the hospital (*T* = 0), his symptoms have been episodic, did not cause significant impact to patient’s quality of life and as such was managed conservatively in the community.

**Fig. 1:**
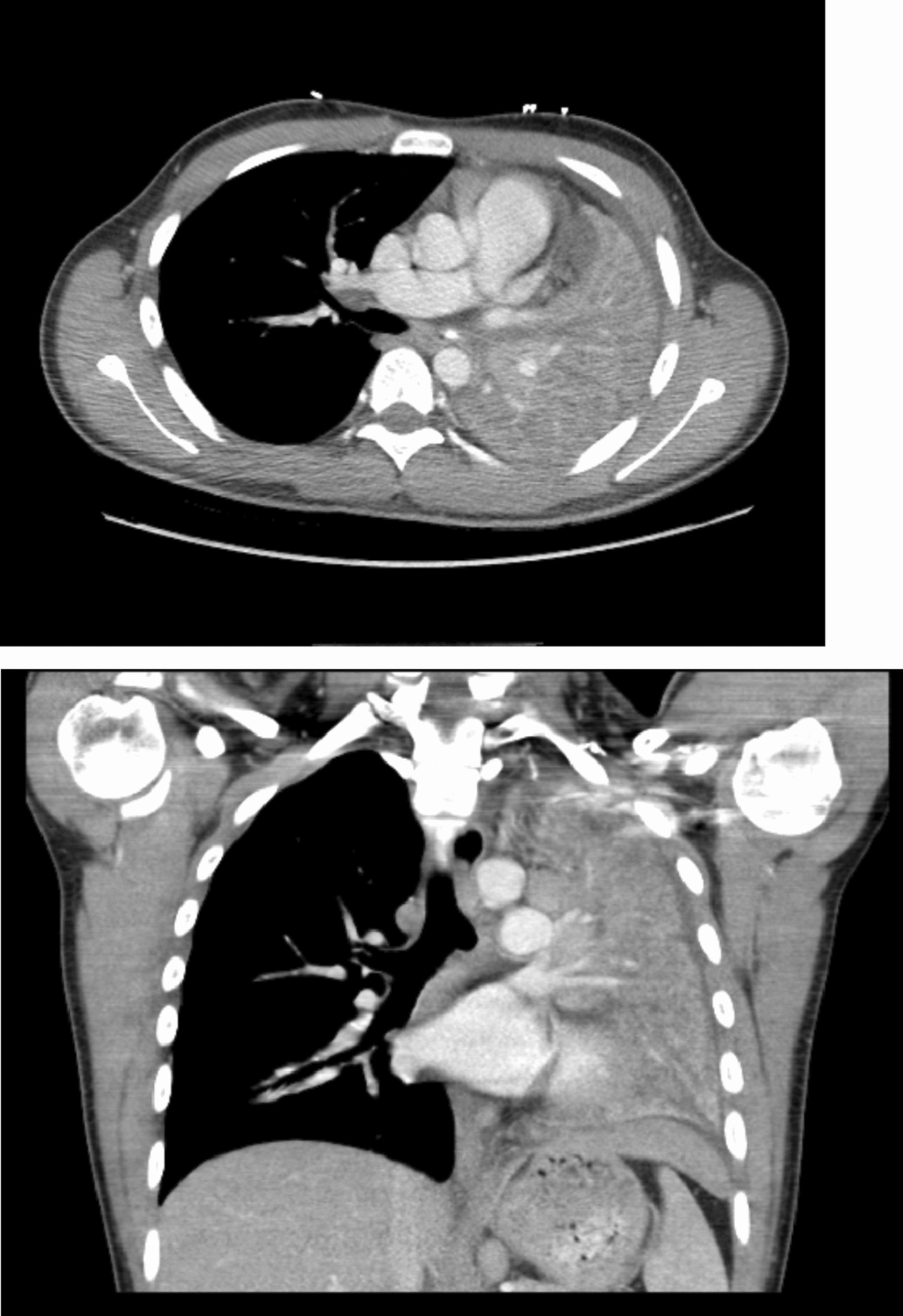
CT showing left bronchial obstruction secondary to tumour compression at presentation

**Fig. 2:**
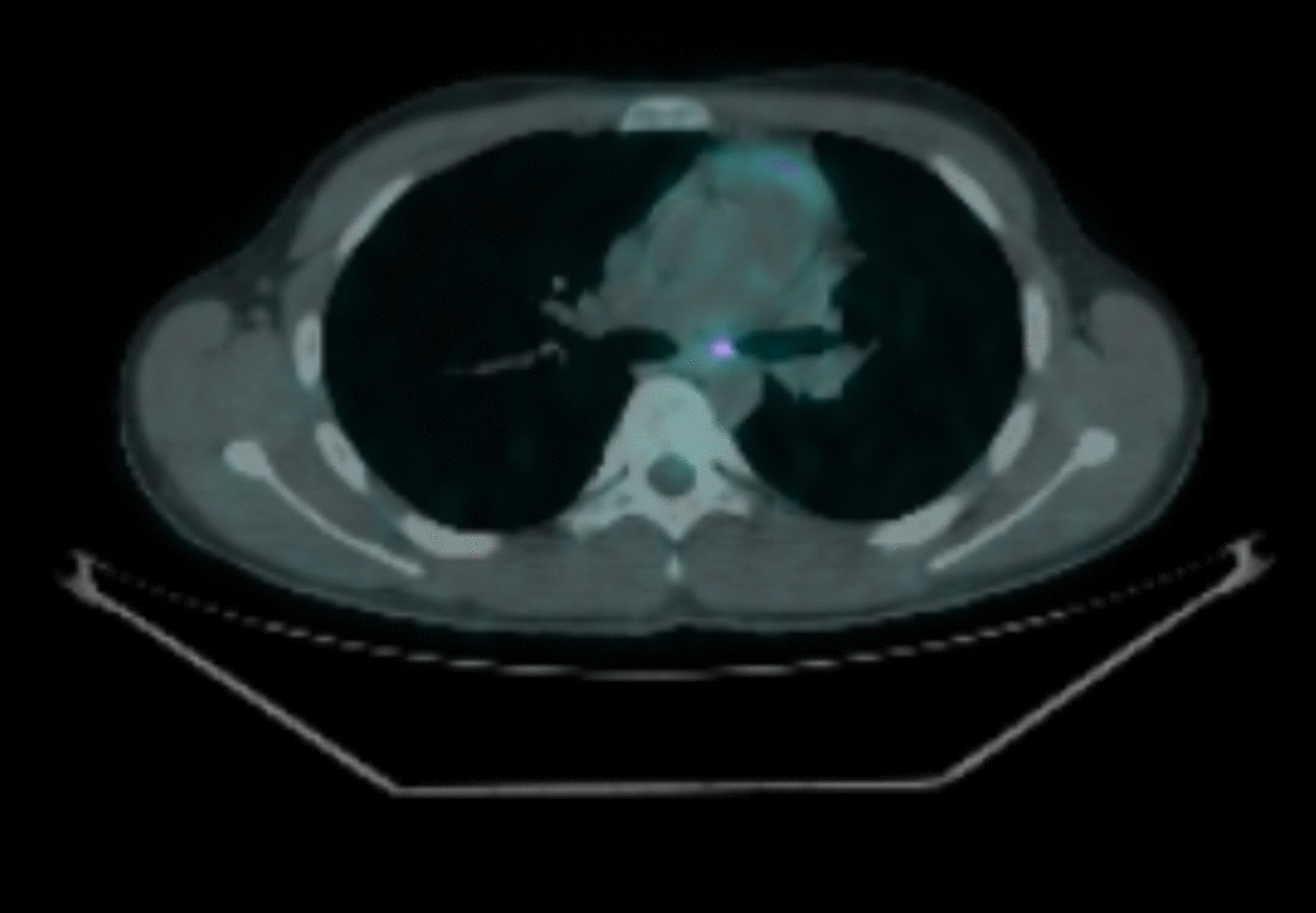
PET-CT showing subcarinal uptake at presentation

During his acute presentation, he was admitted to his local hospital for further investigations (*T* = 0). A CT thorax with contrast showed an area of high density likely representing a tumour at the left main bronchus causing obstruction and collapse secondary to this (Fig. 1 near here). In addition, PET-CT showed an avid solitary calcified subcarinal node of unknown significance (Fig. 2 near here). At bronchoscopy, tumour was visible at the left main bronchus and the initial biopsy was non-diagnostic. Repeat biopsy with rigid bronchoscopy and Nd-Yag laser treatment to intraluminal tumour was performed. Biopsies showed spindle-shaped cells which expressed H-Caldesmon, ALK-1, CK8/18 and CD31. This was in keeping with inflammatory myofibroblastic tumour. Fluorescence in situ hybridization (FISH) confirmed ALK translocation (2p23).

### Treatment and clinical course

The patient was discussed at the Multidisciplinary Team meeting (MDT). Treatment options suggested were to repeat bronchoscopic laser treatment and debulking or Crizotinib, an ALK-inhibitor. The patient was referred by the local Oncologist to the TYA unit at a Tertiary Oncology Centre to discuss this further (*T* = 1 month). As the patient was doing his final year school exams, he was keen to balance the proposed treatment and associated side-effects with his personal plans, including travelling and having a gap-year after his A-levels. Patient was also concerned about the potential side-effects, particularly hypogonadism associated with Crizotinib. He was offered sperm banking but declined it. Other treatment options including surgery, chemotherapy as well as corticosteroids were considered at MDT discussion. It was felt surgery would involve pneumonectomy. Furthermore, a concern was raised that given the extent of tumour extension from the left main bronchus into the carina, there was a strong possibility of a positive margin after pneumonectomy. Furthermore, at presentation he had subcarinal lymphadenopathy on imaging. There was concern of disease recurrence despite pneumonectomy. Subsequent radiology review in MDT showed an interval reduction of mediastinal lymphadenopathy on imaging spontaneously and a period of observation of mediastinal lymph nodes was recommended (*T* = 3 months).

A repeat rigid bronchoscopy five months after initial bronchoscopic intervention showed left main bronchus occlusion (*T* = 5 months). Rigid bronchoscopic dilatation and Nd-Yag laser was applied to tumour regrowth. Over the next two years, the patient had this intervention repeated six times further due to aggressive tumour recurrence.

Nine months following his diagnosis, after careful discussion with his Oncologist, the patient decided to start prednisolone 60 mg once daily (*T* = 9 months). He struggled with side-effects from steroids, including increase in appetite, acne and weight gain. He was weaned off his steroids after a three-month trial period (*T* = 12 months). Patient had modest benefit from corticosteroids at best and required two further therapeutic bronchoscopic procedures during this period. He also developed bronchiectasis due to recurrent airway obstruction and infections. He was referred to Respiratory physicians and started on prophylactic antibiotics (Moxifloxacin). His performance status remained zero.

**Fig. 3:**
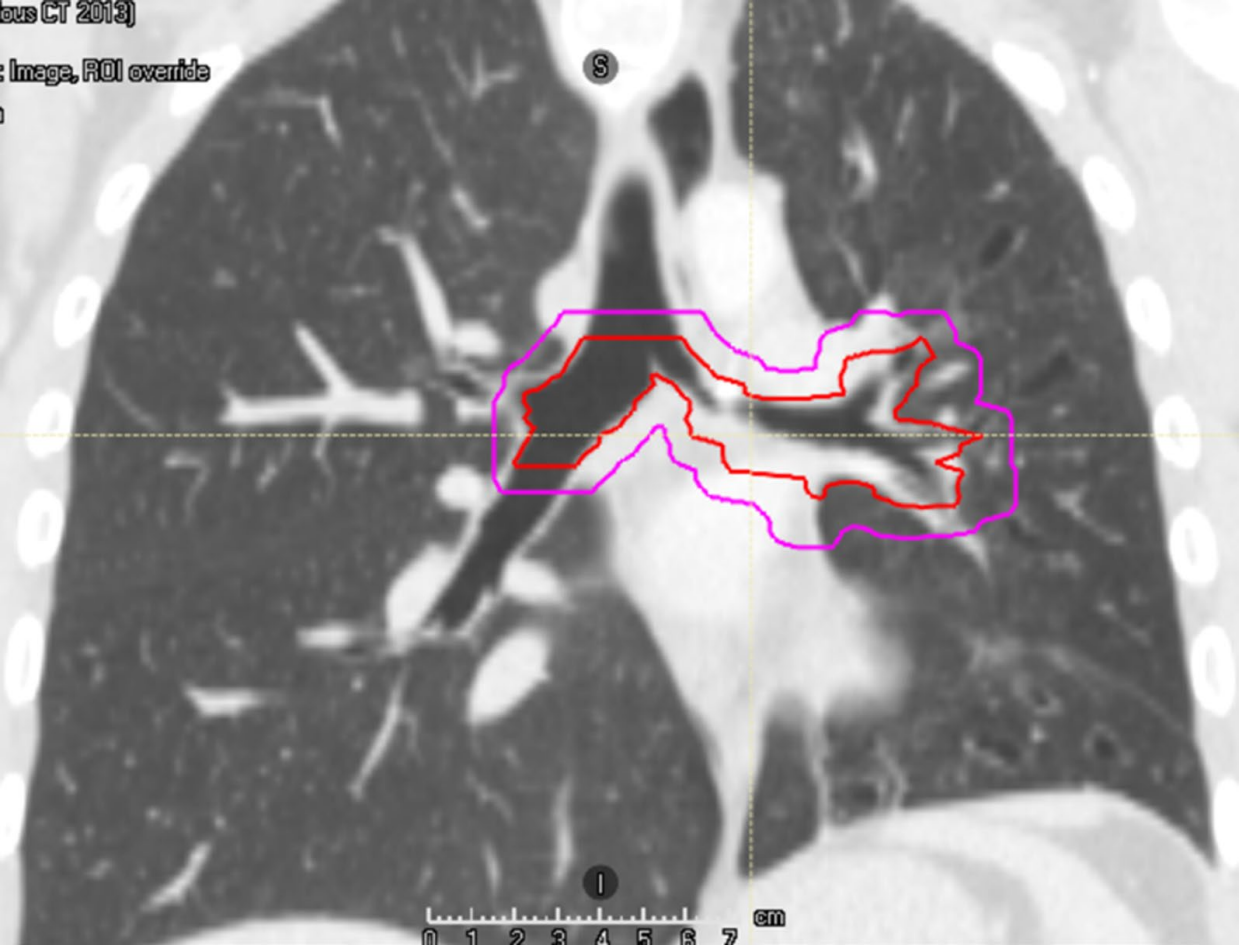
Radiotherapy contours in coronal view, with gross tumour volume in red and planning target volume in magenta. Software: RayStation

**Fig. 4:**
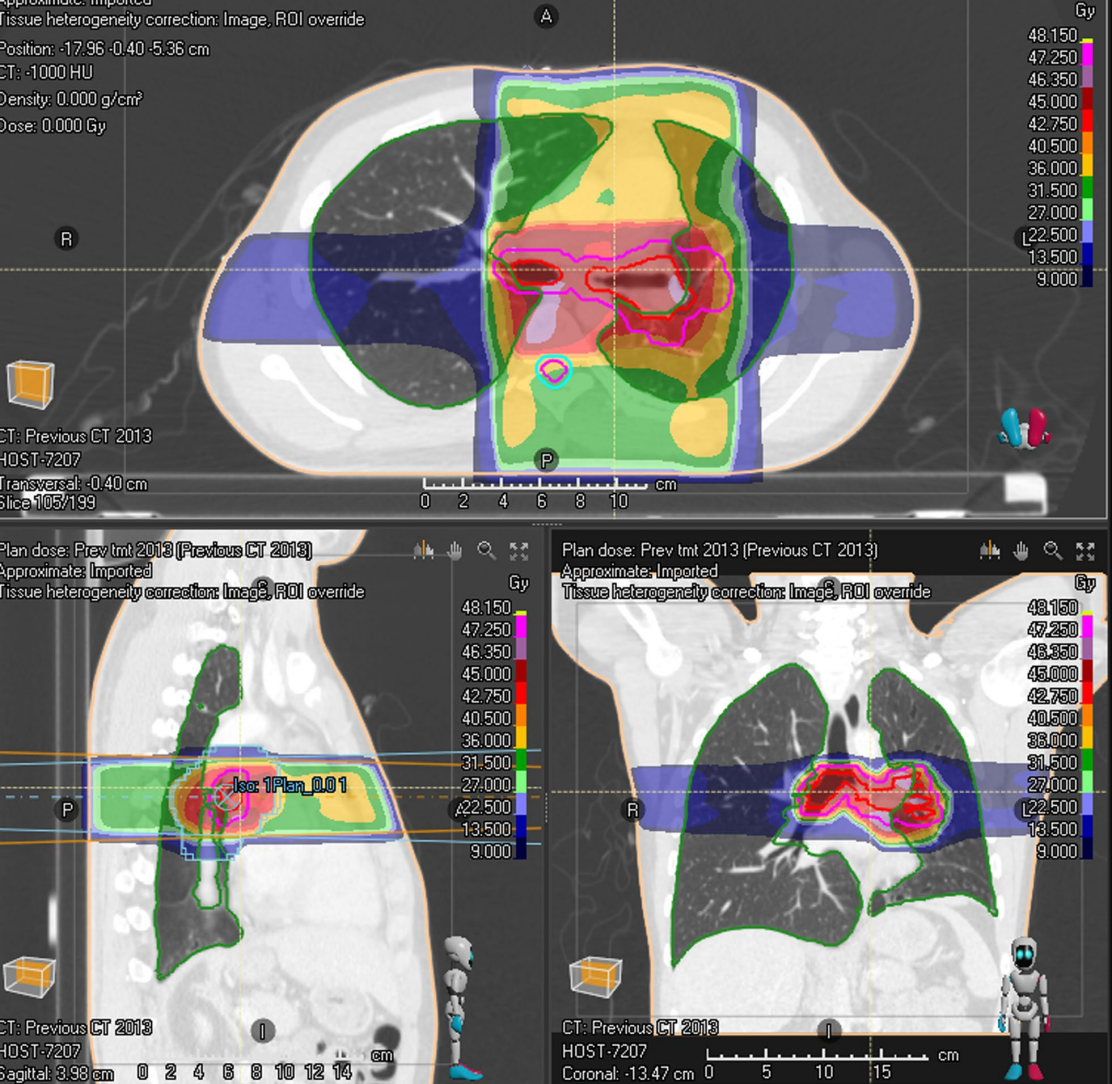
Radiotherapy 3D-conformal plan, with four-field arrangement, anterior and posterior 10 MV beams, and lateral 6 MV beams. Planning software: RayStation

Due to limited treatment options and patient’s reluctance to commence on an ALK inhibitor, he was rediscussed at the MDT and radical radiotherapy was recommended. For radiotherapy planning scan, patient was scanned supine, arms up on chest board, with activated breathing control device. To aid radiotherapy planning, he had bronchoscopic tumour mapping of macroscopic disease to aid radiotherapy delineation. Macroscopic disease was delineated as Gross Tumour Volume (GTV), which included left main bronchus, extending to and including sub-carina superiorly, and proximal right main bronchus as suggested by bronchoscopic findings (Fig. 3 near here). GTV to Planning Target Volume (PTV) margin was 7 mm in all directions. 3D-conformal planning was used and a four-field plan was generated, using anterior and posterior 10 megavoltage (MV) beams and left and right lateral 6MV beams (Fig. 4 near here). He received 45 Gy over 25 fractions over five weeks (*T* = 15 months). He was supported with pulsed steroids (prednisolone 30 mg one week on, one week off) during radiotherapy. This was weaned upon completion of radiotherapy.

Patient was clinically stable following radiotherapy and was able to go abroad for a field trip. He had a bronchoscopic assessment three months following radiotherapy and biopsies showed no sign of IMT (*T* = 18 months). CT scan confirmed an improvement in the left main bronchus patency. Further bronchoscopy four months later showed a patent lumen in the left main bronchus where previously this was completely occluded (*T* = 22 months). Despite this, patient proceeded to developing recurrent chest infections and exacerbation of bronchiectasis. He required multiple courses of antibiotics and was started on prophylactic antibiotics (Azithromycin). He was discussed at the MDT in light of these recurrent infections. Although there was no disease identified on biopsy, the MDT raised concern over an area of increased soft tissue thickening in the left proximal bronchial tree in the latest CT. Due to this radiological finding and the clinical deterioration, it was felt that NSAIDs such as Ibuprofen or Celecoxib could be considered. There was concern over cardiac toxicities associated with Celecoxib. As a result, patient was initiated on Ibuprofen 400 mg TDS one year following radical radiotherapy (*T* = 30 months).

Patient developed good clinical response following this, and bronchoscopic assessment and biopsy six months later confirmed no further disease in the airway (*T* = 36 months). He stopped his NSAIDs within a year (*T* = 42 months) and remained in remission for the last seven years (*T* = 11 years). He has an ECOG performance status of zero, remains fully active. He has since completed a University degree and is now in full-time employment. He continues to have annual clinical follow-ups with chest x-rays.

## Discussion

Pulmonary IMTs are tumours with intermediate malignant potential which tend to recur locally and rarely metastasise. Due to its rare nature, they can often be misdiagnosed as asthma in the community, resulting in long delays before patients receive their diagnosis. These patients often present with recurrent chest infections, which improve following antibiotics. Similar diagnostic challenges present for other uncommon conditions such as pulmonary carcinoids in the community. Young patients with recurrent chest infections and asthma exacerbations not responding to inhalers should be referred to Respiratory Specialists.

Surgical resection remains the treatment of choice for most cases of pulmonary IMT. Bronchoscopic interventions using methods such as laser resection, diathermy, mechanical debulking, cryotherapy have been reported [4, 12, 13]. Recurrence rate can vary from 1.5–5% for pulmonary IMT to 8–25% in single organ extrapulmonary IMTs [5, 14, 15]. It is not uncommon to require repeated bronchoscopic procedures to achieve disease control [12, 16–18].

The patient discussed here had repeated bronchoscopic interventions with Nd-Yag laser for tumour debulking. In inoperable or recurrent IMTs where disease control is not obtained, Crizotinib is a good option for IMTs with ALK translocation [10, 19, 20]. Ceritinib can be considered on progression [21]. The patient was reluctant to start on ALK inhibitor due to potential side-effect of hypogonadism. He was started on a course of corticosteroids and there are several case reports supporting this [6, 7, 22]. Chun *et al*. [7] reports a patient with unilateral solitary IMT who developed local recurrence and received external beam radiotherapy (EBRT) followed by a three-year course of prednisolone. She remains in remission 18 years later. Two other patients who received prednisolone in the series had bilateral pulmonary IMTs had much worse prognosis, dying two and four years after diagnosis [7]. On the other hand, Lee *et al*. [6] reports a case of patient with bilateral pulmonary IMT developing complete response 1 month following corticosteroids. Patient continued to be in remission at 20 months follow-up [6].

The patient reported struggled with Cushingoid side-effects from corticosteroids and wished to consider other local treatment modality. External beam radiotherapy was recommended at this stage. Imperato *et al*. [11] reported two cases of pulmonary IMT receiving 45 Gy in 28 fractions and 43.2 Gy in 24 fractions respectively. The first patient developed contralateral pulmonary IMT three months following this and received EBRT 20 Gy in 5 fractions to contralateral lung [11]. The patient has remained in remission for 11 years since [11]. Hoover *et al*. [23] reported a case treated with EBRT 40.4 Gy in 19 fractions and continued to be in remission 31 months later. Mehta *et al*. [24] reported an unusual case of a 71 year-old with severe COPD treated with 18 Gy in 9 fractions following local recurrence from bronchoscopic resection. A Further 30 Gy in 10 fractions was given six months later due to persistent disease on bronchoscopic examination [24]. Two years on patient continued to have stable disease [24]. Had the patient received radical dose upfront, patient may have developed better disease control, however this was an unusual case presenting in a 71 year-old with fitness precluding radical treatment. Radiotherapy in this particular case was given for symptom control. Imperato *et al*. [11] recommended an EBRT dose of 40–45 Gy in 1.8–2 Gy per fraction. The Patient reported here received 45 Gy in 1.8 Gy per fraction.

NSAIDs in pulmonary IMTs have been utilised and reported in single case reports. Chavez *et al*. [8] reported a case of ALK-negative pulmonary IMT who continued to be in complete response 32 months following starting Celecoxib, a cyclooxygenase-2 (COX-2) inhibitor. The Patient remained on a reduced dose of 200 mg on alternate days [8]. Ramirez *et al*. [25] reported a case treated with Celecoxib for 11 months and four sessions of argon plasma coagulation over the period. She remained in remission six years on [25]. Ghani *et al*. [26] reported a similar case treated with 12 months of Celecoxib and bronchoscopy at eight months showed complete response. Chan *et al*. [27] reported a case with complete response treated with Rofecoxib, another COX-2 inhibitor for eight months. Our patient had no biopsy-proven active disease following radiotherapy. However, NSAIDs were recommended by MDT due to his clinical deterioration. He was started on Ibuprofen due to concerns over the cardiac side-effects of Celecoxib. He developed good clinical response and remained on this for < 12 months. In retrospect, his disease may have been controlled with radiotherapy and NSAIDS played a role in treating his bronchiectasis exacerbation. He continues to be remission seven years on.

### Proposed mechanism for radical radiotherapy and pulse steroids

To our knowledge, this is the first published report of pulmonary IMT treated with radiotherapy and pulse steroids. Short-course steroids is associated with increased mitochondrial biogenesis and enzymatic activity of respiratory chain of immune cells [28]. This enhances immune reactivation and improves anti-tumour response of the immune system following radiotherapy [28–30]. Long-term use of steroids on the other hand can cause abnormal mitochondrial biogenesis [28].

## Conclusions

Pulmonary IMTs are tumours with intermediate malignant potential which tend to recur locally and rarely metastasise. Pulmonary IMTs can behave indolently and usually surgical resection will suffice. If surgical resection is not possible, or the disease behaves more aggressively, then other treatments should be considered. In the case presented, local therapy with bronchoscopic intervention was not sufficient to offer long term disease control which signified aggressive disease, and there was concern over achieving clear margins with radical resection. External beam radiotherapy can offer local control in solitary pulmonary IMTs. ALK-inhibitors often produce excellent and durable response in both localised and extensive IMTs. NSAIDs or corticosteroids should be considered but need to be balanced against its side-effects. Chemotherapy should be reserved for metastatic disease, which clearly exhibits a different tumour biology. Although late relapse is uncommon in pulmonary IMTs, patients should continue to be monitored due to the salvage options available and the favourable outcome in most cases.

## Data Availability

The authors confirm that the data supporting the findings of this study are available within the article [and/or] its supplementary materials.

## References

[1] Coffin CM, Patel A, Perkins S (2001). ALK1 and p80 expression and chromosomal rearrangements involving 2p23 in inflammatory myofibroblastic tumor. Mod Pathol.

[2] Gleason BC, Hornick JL (2008). Inflammatory myofibroblastic tumours: where are we now?. J Clin Pathol.

[3] Coffin CM, Hornick JL, Fletcher CD (2007). Inflammatory myofibroblastic tumor: comparison of clinicopathologic, histologic, and immunohistochemical features including ALK expression in atypical and aggressive cases. Am J Surg Pathol.

[4] Kovach SJ, Fischer AC, Katzman PJ (2006). Inflammatory myofibroblastic tumors. J Surg Oncol.

[5] Coffin CM, Watterson J, Priest JR (1995). Extrapulmonary inflammatory myofibroblastic tumor (inflammatory pseudotumor). A clinicopathologic and immunohistochemical study of 84 cases. Am J Surg Pathol..

[6] Lee MH, Lee HB, Lee YC (2011). Bilateral multiple inflammatory myofibroblastic tumors of the lung successfully treated with corticosteroids. Lung.

[7] Chun YS, Wang L, Nascimento AG (2005). Pediatric inflammatory myofibroblastic tumor: anaplastic lymphoma kinase (ALK) expression and prognosis. Pediatr Blood Cancer.

[8] Chavez C, Hoffman MA (2013). Complete remission of ALK-negative plasma cell granuloma (inflammatory myofibroblastic tumor) of the lung induced by celecoxib: a case report and review of the literature. Oncol Lett.

[9] Bertocchini A, Lo Zupone C, Callea F (2011). Unresectable multifocal omental and peritoneal inflammatory myofibroblastic tumor in a child: revisiting the role of adjuvant therapy. J Pediatr Surg.

[10] Butrynski JE, D'Adamo DR, Hornick JL (2010). Crizotinib in ALK-rearranged inflammatory myofibroblastic tumor. N Engl J Med.

[11] Imperato JP, Folkman J, Sagerman RH (1986). Treatment of plasma cell granuloma of the lung with radiation therapy. A report of two cases and a review of the literature. Cancer.

[12] Conforti S, Bonacina E, Ravini M (2007). A case of fibrous histiocytoma of the trachea in an infant treated by endobronchial ND:YAG laser. Lung Cancer.

[13] Iyer A, Radonic T, Heukamp LC (2021). Inflammatory myofibroblastic tumour of the central airways: treatment and molecular analysis. ERJ Open Res.

[14] Yousem SA, Tazelaar HD, Manabe T, Travis WD, Brambilla E, MullerHermelink HK (2004). Inflammatory myofibroblastic tumour. Pathology and genetics of tumours of the lung, pleura, thymus and heart.

[15] Janik JS, Janik JP, Lovell MA (2003). Recurrent inflammatory pseudotumors in children. J Pediatr Surg.

[16] Brodlie M, Barwick SC, Wood KM (2011). Inflammatory myofibroblastic tumours of the respiratory tract: paediatric case series with varying clinical presentations. J Laryngol Otol.

[17] Wang H, Zhang N, Tao M (2012). Application of interventional bronchoscopic therapy in eight pediatric patients with malignant airway tumors. Tumori.

[18] Andrade FM, Abou-Mourad OM, Judice LF (2010). Endotracheal inflammatory pseudotumor: the role of interventional bronchoscopy. Ann Thorac Surg.

[19] Lovly CM, Gupta A, Lipson D (2014). Inflammatory myofibroblastic tumors harbor multiple potentially actionable kinase fusions. Cancer Discov.

[20] Schöffski P, Sufliarsky J, Gelderblom H (2018). Crizotinib in patients with advanced, inoperable inflammatory myofibroblastic tumours with and without anaplastic lymphoma kinase gene alterations (European Organisation for Research and Treatment of Cancer 90101 CREATE): a multicentre, single-drug, prospective, non-randomised phase 2 trial. Lancet Respir Med.

[21] Mansfield AS, Murphy SJ, Harris FR (2016). Chromoplectic TPM3-ALK rearrangement in a patient with inflammatory myofibroblastic tumor who responded to ceritinib after progression on crizotinib. Ann Oncol.

[22] Doski JJ, Priebe CJ, Driessnack M (1991). Corticosteroids in the management of unresected plasma cell granuloma (inflammatory pseudotumor) of the lung. J Pediatr Surg.

[23] Hoover SV, Granston AS, Koch DF (1977). Plasma cell granuloma of the lung, response to radiation therapy: report of a single case. Cancer.

[24] Mehta J, Desphande S, Stauffer JL (1980). Plasma cell granuloma of the lung: endobronchial presentation and absence of response to radiation therapy. South Med J.

[25] Ramirez IA, Rubalcava NS, Mychaliska GB (2020). Recurrent endobronchial inflammatory myofibroblastic tumors: novel treatment options. Pediatr Pulmonol.

[26] Ghani S, Desai A, Pokharel S (2015). Pneumonectomy-sparing NSAID therapy for pulmonary inflammatory myofibroblastic tumor. J Thorac Oncol.

[27] Chan PW, Omar KZ, Ramanujam TM (2003). Successful treatment of unresectable inflammatory pseudotumor of the lung with COX-2 inhibitor. Pediatr Pulmonol.

[28] Kokkinopoulou I, Moutsatsou P (2021). Mitochondrial glucocorticoid receptors and their actions. Int J Mol Sci.

[29] Taghizadeh-Hesary F, Houshyari M, Farhadi M (2023). Mitochondrial metabolism: a predictive biomarker of radiotherapy efficacy and toxicity. J Cancer Res Clin Oncol.

[30] Houshyari M, Taghizadeh-Hesary F (2023). Is mitochondrial metabolism a new predictive biomarker for antiprogrammed cell death protein-1 immunotherapy?. JCO Oncol Pract.

